# Increased numbers of P63-positive/CD117-positive cells in advanced adenoid cystic carcinoma give a poorer prognosis

**DOI:** 10.1186/1746-1596-7-119

**Published:** 2012-09-10

**Authors:** Quan Zhou, Hong Chang, Hongkai Zhang, Yiding Han, Honggang Liu

**Affiliations:** 1 Department of Pathology, Beijing Tongren Hospital, Capital Medical University, Dongjiaominxiang No. 1 Dongcheng District, Beijing, China; 2 Department of Pathology, Beijing Shijitan Hospital, Capital Medical University, Tieyilu No. 10, Haidian District, Beijing, China; 3 Department of Pathology, Beijing Fuxing Hospital, Capital Medical University, Fuxingmenwaidajiejia No. 20, Beijing, China

**Keywords:** Carcinoma, Adenoid cystic, CD117, Myoepithelium, Prognosis

## Abstract

**Objectives:**

This study consisted of two parts. One part was to analyze the survival rates of adenoid cystic carcinoma (ACC) in Chinese and explain the difference between our data and the literature. The other was to analyze the relationship between the expression of CD117 and the histological grade and the prognosis.

**Methods:**

A retrospective study of 80 ACC patients was performed. Clinical data were collected, and p63, CD117 were detected by immunohistochemical staining.

**Results:**

Eighty patients received follow-ups 3 to 216 months after initial diagnosis. ACC occurred in the lacrimal gland (26.3%, n = 21), nasal cavity and parasinus (33.8%, n = 27) and other sites (40.0%, n = 33). The 5-year and 10-year survival rates were 66.41% and 10.16%, respectively. Over expression of CD117 was detected in p63-negative cells in 94.3% of cases and in p63-positive cells in 45.8%. The expression of CD117 in p63-positive cells was significantly associated with the histological grade (P<0.001) and prognosis (P = 0.037) in patients in the advanced stage.

**Conclusions:**

ACC had a good 5-year survival but poor 10-year survival in Chinese, which differed from the occidental data. More p63+/CD117+ cells were associated with a higher histological grade and poorer outcome.

**Virtual slides:**

The virtual slide(s) for this article can be found here: http://www.diagnosticpathology.diagnomx.eu/vs/1701457278762097

## Background

Adenoid cystic carcinoma (ACC), first described in the 1850 s, is an uncommon malignant tumor that accounts for less than 5 percent of head-and-neck cancers [1]. ACC is characteristic for its indolent nature and its propensity for late distant metastases. Previous studies have shown CD117 to be positive in most ACC; however, no study has focused on the relationship between expression, histological grade and prognosis.

## Methods and materials

From 2005 to 2012, a total of 125 ACC patients were seen by the department of pathology in Beijing Tongren Hospital. All samples were obtained after patients had provided written, informed consent. Of these patients, 80 received substantial follow-ups between 3 to 216 months after initial diagnosis. According to histological patterns, tumors were divided into 4 grades: Grade 1 was tubular/cribriform; Grade 2 was solid type of less than 30%; Grade 3 was solid type equal to or more than 31%; and Grade 4 showed high grade transformation. The patients were divided into 2 stages. The early stage meant that the cancer was limited to the organ in which it began, without evidence of spreading. The advanced stage meant that the cancer had spread beyond the primary site to lymph nodes or nearby organs and tissues or distant organs.

### Immunohistochemical staining

CD117 (polyclonal rabbit; dilution 1:400; Dako, Carpintera, CA, USA) and P63 (4A4; dilution 1:100; Dako, Carpintera, CA, USA) were detected in all cases on serial sections by immunohistochemical staining. These were performed on 4 μm thick unstained sections cut from representative formalin-fixed paraffin-embedded blocks by the avidin-biotin-peroxidase complex technique.

### Statistical analysis

Statistical tests were performed using the SPSS software package (12.0, Chicago, IL). Significances were assessed with log-rank statistics, or using the chi-square test. The cox proportional-hazards regression was used to analyze the effect of several risk factors on survival.

## Results

**Figure 1:**
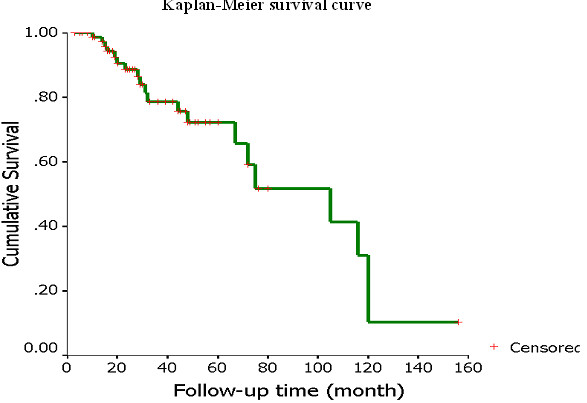
Kaplan–Meier survival curves

Patient characteristics are listed in Table 1. The study population consisted of 38 males and 42 females. Median age was 47.5 years (range, 8–81 years). The subsites included the nasal cavity and paranasal sinuses (n = 27), the lacrimal gland (n = 21) and other (n = 32). The “other” category included the major salivary gland, trachea, ear and tongue. All patients were classified as grade 1 (67.5%, n = 54), grade 2 (15.0%, n = 12), grade 3 (12.5%, n = 10), or grade 4 (5.0%, n = 4). Seventy-six cases were eventually divided into early stage (35%, n = 28) and advanced stage (60%, n = 48). Four cases failed to be classified due to lack of sufficient information. The 1-year, 2-year, 5-year and 10-year rate of overall survival was 88.89%, 79.77%, 66.41% and 10.16%, respectively (Figure 1). Survival was not associated with primary sites (P = 0.885), sex (P = 0.838), or age (P = 0.545). Twenty-two cases (27.5%) had distant metastases. The earliest metastasis predated the primary tumor. The latest was detected 216 months after the initial diagnosis. Median latency was 42.3 months. The most common sites for metastases were lung (12), brain (9), and bone (6). Liver (3) and skin (1) were also present. The following predictors were analyzed as prognostic variables: sex, age, recurrence, the site of metastasis, the subsite of initial tumor, histological grade, surrounding involvement, and nerve and vessel invasion. Only histological grade was significantly associated with prognosis (P = 0.031). Higher grade was related to poorer prognosis.

**Table 1:** Patient characteristics

**Parameter**	**n (%)**
Gender	
Male	38 (47.5)
female	42 (52.5)
Subsite	
Lacrimal gland	21 (26.3)
Nasal cavity and paranasal sinuses	27 (33.8)
Other	32 (40.0)
Recurrence	29 (36.3)
Metastasis	
Distant	22 (27.5)
Surrounding involvement	51 (63.4)
Nerve and vessel involvement	52 (65.0)
Histological grade	
Grade 1	53 (66.3)
Grade 2	14 (17.5)
Grade 3	9 (11.3)
Grade 4	4 (5.0)

**Figure 2:**
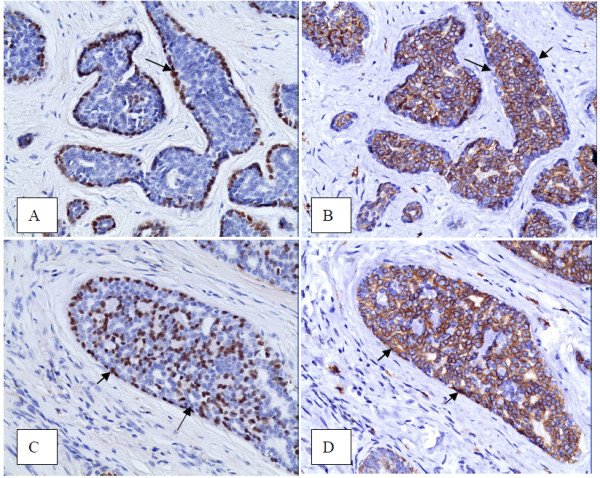
**Immunostaining patterns for p63 and CD117.A** and **B**, and **C** and **D** are 2 couples of serial sections, respectively. **A** and **C** show immunostaining for p63. **B** and **D** show staining for CD117. Compared with **A**, **B** showed p63-/CD117+ cells (arrows). Compared with **C**, **D** showed p63+/CD117+ cells (arrows)

ACC consisted of 2 types of tumor cells. They were p63-positive cells and p63-negative cells. CD117 was positive in p63-negative cells in 94.3% of cases and was not associated with the histological grade (P = 0.566) or prognosis, regardless of being in the early or advanced stage (Figure 2A and B). CD117, however, was positive in p63-positive cells in 45.8% of cases. P63+/CD117+ cells were not associated with neither grade nor prognosis in the early stage. They were, however, significantly associated with grade (P<0.001) and prognosis (P = 0.037) in the advanced stage (Figure 2C and D).

## Discussion

ACC is well-known for its indolent growth but common recurrence and late distant metastasis. Although the 5-year survival rate is high, 10-year and 15-year survival markedly decreased. Hence, it is classified as “high risk” by the World Health Organization [2]. The classic treatment is complete resection followed by postsurgical radiotherapy. In our study, 3 of 80 patients gave up surgery on initial diagnosis and underwent radiotherapy alone. The tumor developed gradually. The other 77 cases underwent surgery and postsurgical radiotherapy but no chemotherapy. The 5-year and 10-year survival was 66.41% and 10.16%, respectively, results which vary greatly from some reported literature.

In 2005, the World Health Organization reported an extremely low survival rate of ACC. Five-year and 10-year survival rates of ACC in the salivary gland were 35% and 10%, respectively. Ten-year survival in the sinonasal subsite was 7%. The 10-year survival was similar to that in our data, but the 5-year survival was much lower. It was speculated that the cited data came from earlier literature (1974, 1978, 1990), when both the treatment and patient health care were different from today, which eventually caused difference in 5-year survival [1,3,4]. However, ACC is characterized by endless growth and late distant metastasis. It is radiosensitive but cannot be cured by radiation alone. When surgery is unavailable due to recurrence or metastasis, the outcome is poor. This is why the 10-year survival rate was similarly low.

However, Ellington CL et al. has reported an extremely high survival of ACC in 2012, with the 5-year, 10-year and 15-year survival being 90%, 80% and 69%, respectively [5]. Distant metastasis was detected in 11.57% of cases. The data were based on a study of 3026 cases in America between 1973 and 2003. In addition, Gomez DR et al., in 2008, reported the 5-year and 10-year survival was 87% and 65%, respectively [6]. Distant metastasis was detected in 34% of cases. The study was based on 59 cases. The differences between their data and the present study were analyzed in terms of the following: (1) the outcome for patients with ACC in the lacrimal gland and sinonasal subsite was poorer than those with ACC in the salivary gland. Although our study did not reveal that survival was associated with the subsite, it was reported that 5-year survival for patients with sinonasal ACC was from 50% to 62.9% [7,8]. Lacrimal gland ACC and paranasal sinus ACC accounted for 60.0% in our data, 42.4% in Gomez DR’s and 5.12% in Ellington CL’s. More patients with lacrimal gland and sinonasal ACC decreased our survival. (2) There were more patients (60%) in advanced stage in our cohort, compared with Ellington’s (47.95%) and Gomez’s (47.5%). (3) Females had better survival outcomes, as concluded by Ellington CL and Lloyd [9]. The male-to-female ratio was 1:1.1 in our data, 1:1.9 in Gomez DR’s and 1:1.4 in Ellington CL’s. Unfortunately, the relationship between sex and survival was not confirmed in our study. (4) None of our patients received chemotherapy, whereas some patients of the other studies did, including cisplatin, 5-fluorouracil and anthracyclines. Although chemotherapy showed inconsistent results, some patients did benefit from it. (5) Higher grade related to poorer outcome. 17.6% of cases in our study were grade 3 or higher. The other two studies did not list the histological grade; therefore, the patients might be in different grades which would eventually cause differences in outcomes. (6) All patients in our study were Chinese. Asian and Pacific Islanders accounted for only 7.77% in Ellington CL’s study and none in Gomez DR’s. Race might play a role. (7) The social and economic position should not be ignored in medical behavior. Despite explanations for the differences, further studies are required to confirm that a “high risk” carcinoma can still have an excellent patient outcome.

CD117 (KIT) is a type III receptor tyrosine kinase operating in cell signal transduction in several cell types. KIT is activated by binding of its ligand, which leads to a phosphorylation cascade ultimately activating various transcription factors in different cell types. CD117 has been detected in many normal cells and tumor cells [10]. Previous studies have shown that 78% to 100% of ACC expressed CD117 [10-15]. But only Mino M et al. briefly discussed the relationship between the pattern of immunostaining and the different histological subtypes. Mino’s data were based on a cohort of 66 cases. The data revealed that the solid subtype of ACC showed a mostly diffuse pattern and the tubular and cribriform subtypes demonstrated CD117 expression primarily within the luminal epithelial cell layer, which was p63 negative. The authors, however, did not discuss CD117 expression in p63-positive cells.

Our study focused on the immunostaining patterns, not only in p63-negative cells but also in p63-positive cells, and compared the patterns with histology grades and prognosis. Our study revealed that the P63-/CD117+ pattern was not associated with histological grade or prognosis in either stage. Interestingly, the P63+/CD117+ pattern was not related to grade or prognosis in the early stage. It was, however, significantly related to grade and prognosis in the advanced stage. Increased numbers of P63+/CD117+ cells meant a higher histological grade and a poorer prognosis. The current theory was that the organ stem cell with pluripotent differentiation capability would unidirectionally differentiate toward myoepithelia (p63-positive cells) or glandular epithelia (p63-negative cells) in ACC. Since myoepithelia has cytoplastic smooth muscle fibril, and smooth muscle cells are constantly negative for CD117, it is inferred that the p63-positive/CD117-positive cells are tumor cells with less differentiation toward myoepithelia. Once the differentiation is extensively insignificant (meaning p63-positive cells remarkably decrease or are absent), high grade transformation will be present, which was once called dedifferentiation.

Our study also revealed the different significance of p63+/CD117+ cells in early stage and advanced stage, respectively. In addition to the non-relationship between stage and prognosis, it was thought that ACC in different stages were heterogenous.

## Conclusion

Our study revealed differences in survival rates of ACC between Chinese and occidental data, with more studies expected to further explain the difference. This study revealed the relationship between the patterns of CD117 expression and histological grades and prognosis in the advanced stage. In addition to traditional histological subtypes and novel p-AKT [15], the expression of CD117 could help to assess prognosis.

## Competing interests

The authors declare that we haven’t any financial competing interests.

## Authors’ contributions

ZQ participated in the design of the study and drafted the manuscript. HY carried out the immunoassays. ZH participated in the sequence alignment. LH and CH conceived of the study, and participated in its design and coordination and helped to draft the manuscript. All authors read and approved the final manuscript.

## Statements

All the authors are in agreement as to the contents of this article and agree to send the article for review and wish for it to be published in *Diagnostic Pathology*. This study conformed to regulations concerning the privacy rights of patients. The authors have nothing else to declare.
